# AdjuvareDB: A comprehensive database for candidate adjuvant compendium in clinic

**DOI:** 10.1002/ctm2.1669

**Published:** 2024-04-24

**Authors:** Dekang Ren, Jiaming Jin, Shizheng Xiong, Daoliang Xia, Xinmiao Zhao, Haochuan Guo, Xueni Yang, Jiafeng Yu, Tingming Liang, Li Guo

**Affiliations:** ^1^ State Key Laboratory for Organic Electronics and Information Displays, Institute of Advanced Materials (IAM), Nanjing University of Posts and Telecommunications Nanjing China; ^2^ Jiangsu Key Laboratory for Molecular and Medical Biotechnology, School of Life Science, Nanjing Normal University Nanjing China; ^3^ Shandong Provincial Key Laboratory of Biophysics, Institute of Biophysics, Dezhou University Dezhou China

1

Dear Editor,

Adjuvants are important components of vaccination, stimulating and enhancing the strength and duration of immune responses.^1^ To provide a comprehensive compendium of candidate adjuvants, we developed an integrated database, AdjuvareDB (http://tmliang.cn/adjuvaredb), containing composition, function and other features of 331 potential adjuvants (Figure 1). Of these, candidate genetic adjuvants, maybe pivotal adjuvant components, were provided primary pan‐cancer analysis to understand the molecular features via a multi‐omics approach. The user‐friendly integrated AdjuvareDB is a versatile and extensible tool, which will contribute to further clinical application in vaccine and immunotherapy research.

**FIGURE 1:**
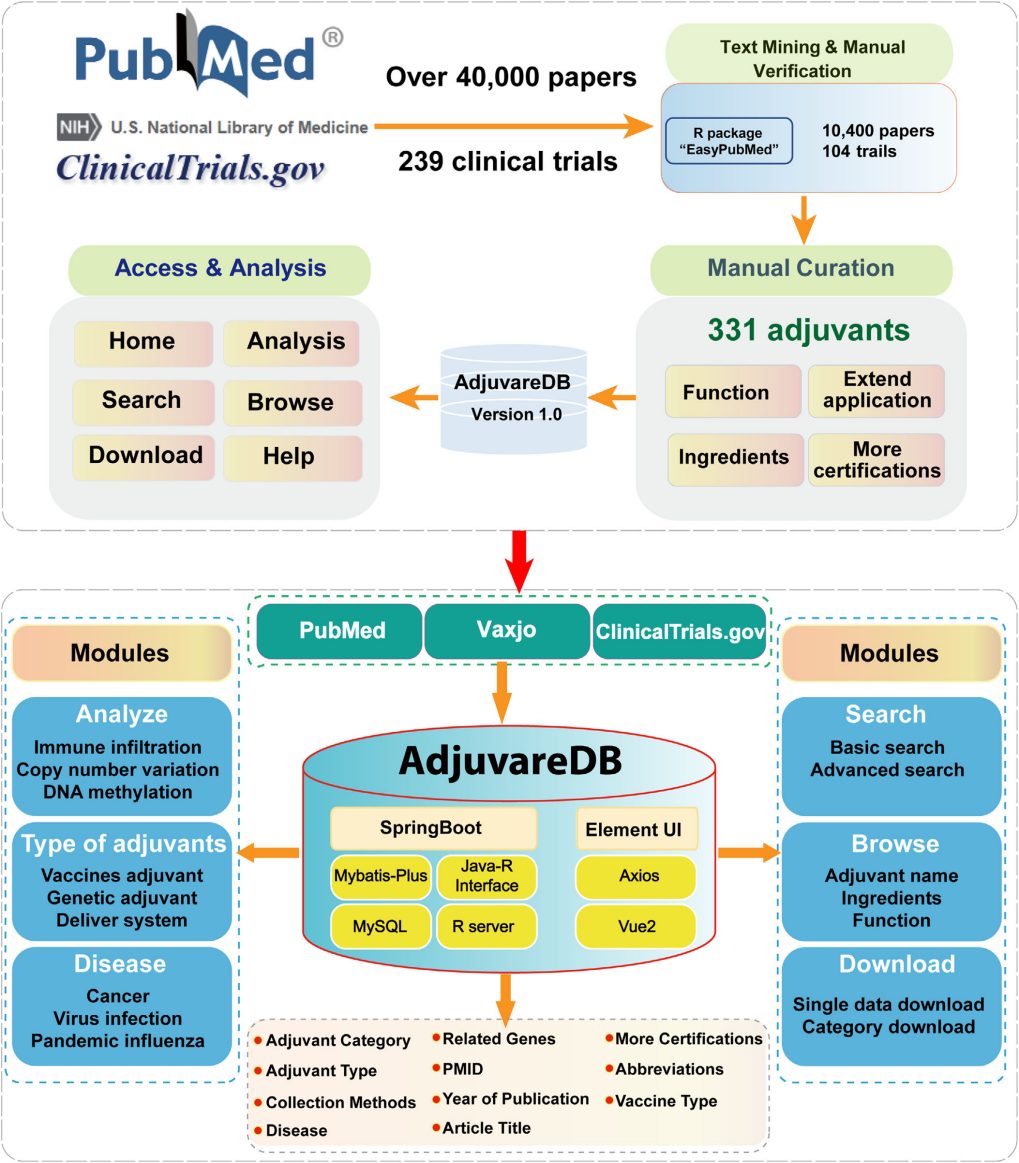
The schematic workflow of data collection and main frame of AdjuvareDB.

Significant adjuvant‐related applications over the past decades have included hepatitis B vaccines, pericoronitis vaccines, and cancer vaccines.^2^, ^3^ Adjuvants are receiving increasing attention as a strategy for immunotherapy, particularly as a key to solving the problems faced by cancer vaccines.^4^, ^5^ A comprehensive understanding of the process of antigen‐adjuvant immunization, the classes of adjuvants available, the molecular targets associated with adjuvants in the innate immune system, will help to identify novel adjuvants, potential immune agonists, novel adjuvant composition schemes, and therapeutic targets.

To provide the most comprehensive information on adjuvants and contribute to the promising application of adjuvants in immunotherapy, we collected adjuvants from published papers and databases for relevant adjuvants in vaccines and novel materials as adjuvants in delivery systems (Figure 1). Adjuvants can be broadly characterized as adjuvant components in vaccines as well as immune adjuvants in immunotherapy based on the ingredients and function, and we selected representative adjuvant regimens and annotated them according to their characteristics, such as chemokines and cytokines among potential genetic adjuvants, immune agonists, and the classical adjuvant aluminum salts (Figure 2A). To demonstrate the clinical use of adjuvants, therapeutic cancer vaccines, a therapeutic combination of vaccines and immunotherapy,^6^, ^7^ were also collected from therapeutic cancer vaccine‐related trials on ClinicalTrials.gov (https://classic.clinicaltrials.gov/), and 27 adjuvants were summarized in 104 therapeutic cancer vaccines (Figure 2B‐C). Furthermore, another 14 adjuvants were obtained from Vaxjo.^8^ Thus, a total of 331 candidate adjuvants were screened and obtained, and the relevant contents were summarized, including source literatures, type of vaccine to which they belonged, the direction of application, composition, function and effect. Based on the integration of information, we have compiled a variety of vaccine design options for cancer vaccines, such as mRNA vaccines, peptide vaccines, and dendritic cell‐based vaccines. All of these were presented in AdjuvareDB (a MySQL database developed utilizing the Spring architecture, Figure 1). Among the collected adjuvants, some potential genetic adjuvants, such as pattern recognition receptors, playing a key role in immune activation and enhancement. These genetic adjuvants were queried by GeneCards,^9^ and 76 genes with a potential role in adjuvant properties, were identified. These genes may also have critical roles in tumorigenesis (Figure 3), and many of them showed significant correlations with immune cells across different cancers, indicating the important roles in immune and cancer processes. In order to further understand the detailed molecular features in tumorigenesis, candidate genetic adjuvants were provided primary pan‐cancer analysis via a multi‐omics approach in AdjuvareDB.

**FIGURE 2:**
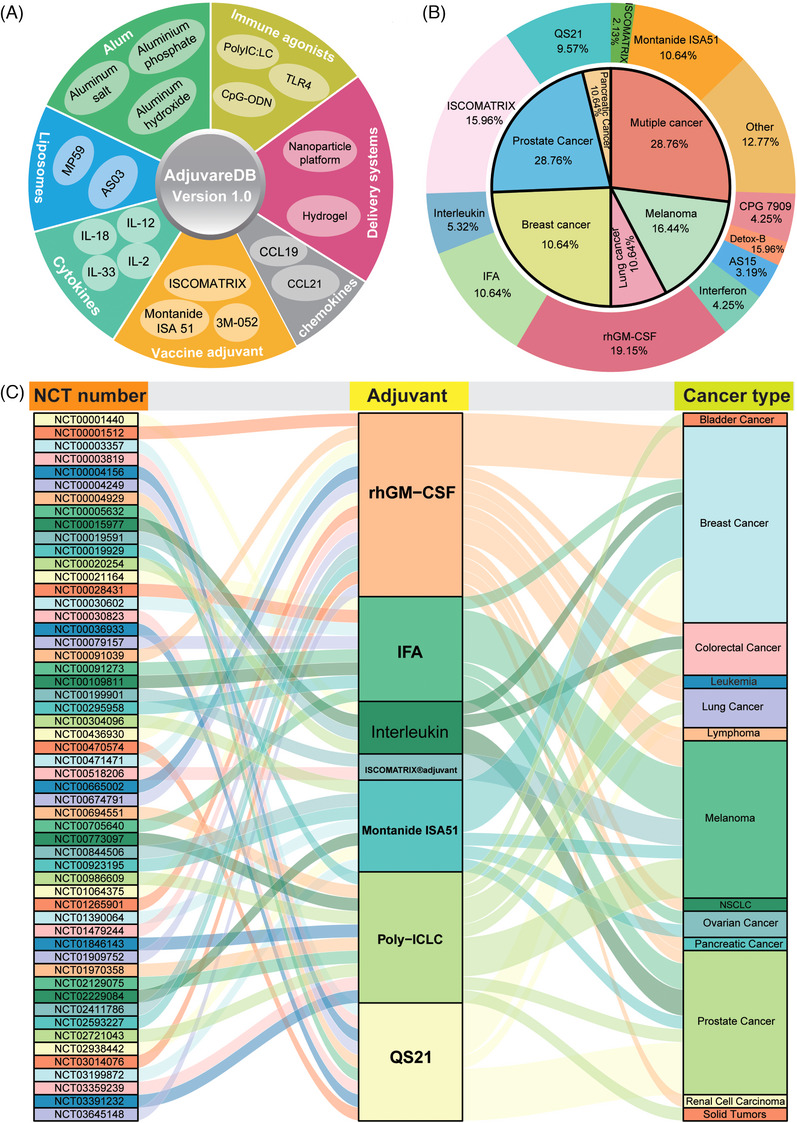
The primary analysis of candidate adjuvants. A. The annotation of candidate adjuvants in AdjuvareDB. B. The distribution of adjuvant and cancer application types based on 104 clinical trial protocols for therapeutic cancer vaccines. C. The alluvial plot of adjuvants involved in therapeutic cancer vaccine trial protocols and the types of cancers targeted, as obtained from ClinicalTrials.gov (https://classic.clinicaltrials.gov/), showing the relationships of National Clinical Trial (NCT) number, adjuvant name, and cancer type among the high frequency of 7 adjuvants used in the trials. Abbreviations: rhGM‐CSF, recombinant human granulocyte‐macrophage colony‐stimulating factor; IFA, Incomplete Freund's Adjuvant; QS21, Quillaja saponaria Molina; CpG‐ODN, cytosine‐phosphodiester‐guanine‐oligonucleotide; Poly IC: LC, Poly‐L‐lysine and carboxymethyl cellulose; NSCLC, Non−Small Cell Lung Carcinoma.

**FIGURE 3:**
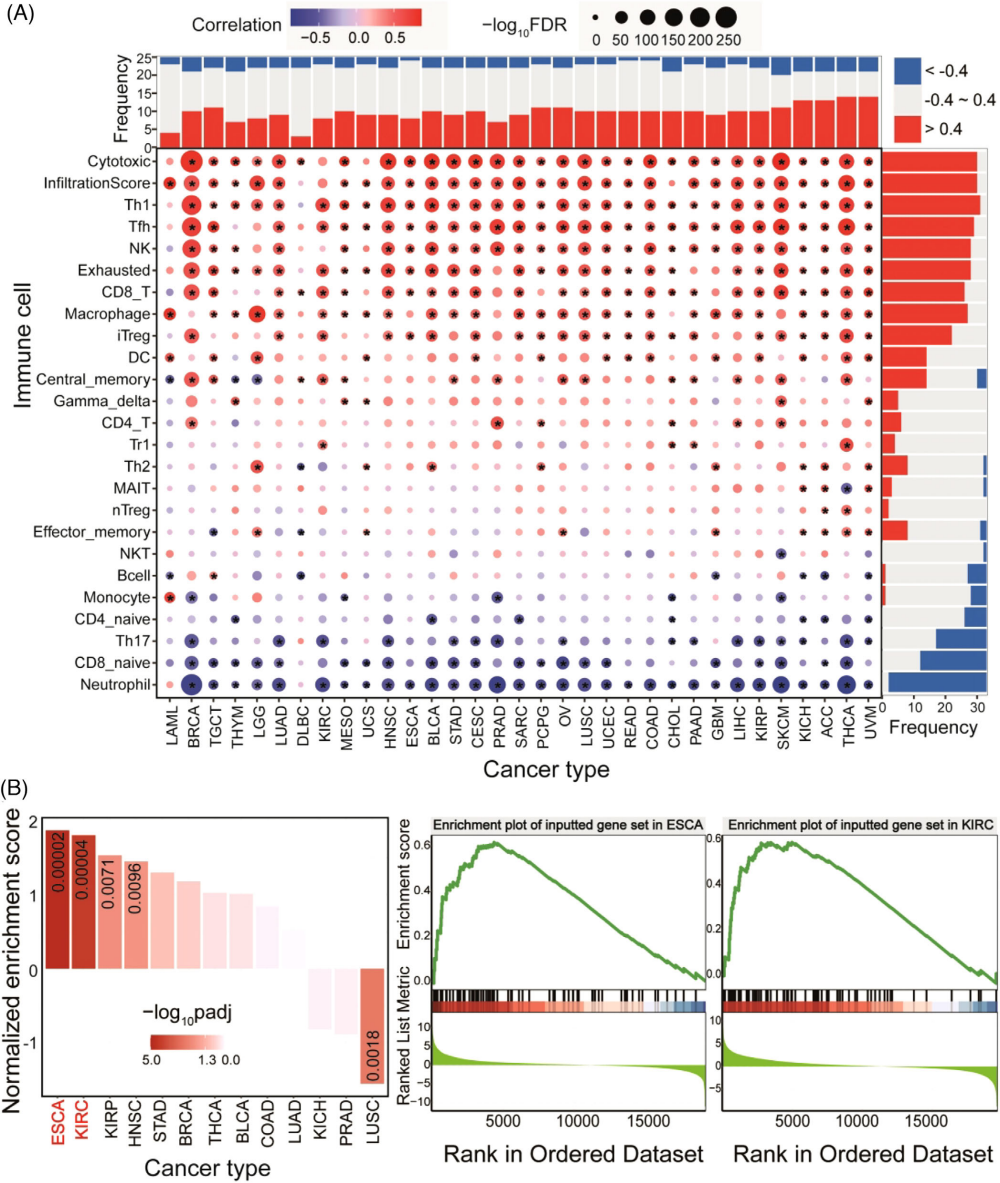
Primary analysis of gene set indicates the roles in tumorigenesis. A. The distributions of the associations between immune cell infiltration and expression level of the gene set identified as potential genetic adjuvants. * indicates significant correlations, r > 0.40 or r < ‐0.40 and FDR < 0.05. B. Bar plot shows distribution of enrichment score via GSEA (Gene Set Enrichment Analysis) in pan‐cancer based on the get set. Significant padj values are presented for some specific cancer types. The right images show enrichment plots in ESCA and KIRC. *Abbreviations of cancers*: ACC, Adrenocortical carcinoma; BLCA, Bladder Urothelial Carcinoma; BRCA, Breast invasive carcinoma; CESC, Cervical squamous cell carcinoma and endocervical adenocarcinoma; CHOL, Cholangiocarcinoma; COAD, Colon adenocarcinoma; DLBC, Lymphoid Neoplasm Diffuse Large B‐cell Lymphoma; ESCA, Esophageal carcinoma; GBM, Glioblastoma multiforme; LGG, Glioma; HNSC, Head and Neck squamous cell carcinoma; KICH, Kidney Chromophobe; KIRC, Kidney renal clear cell carcinoma; KIRP, Kidney renal papillary cell carcinoma; LAML, Acute Myeloid Leukemia; LGG, Brain Lower Grade Glioma; LIHC, Liver hepatocellular carcinoma; LUAD, Lung adenocarcinoma; LUSC, Lung squamous cell carcinoma; MESO, Mesothelioma; OV, Ovarian serous cystadenocarcinoma; PAAD, Pancreatic adenocarcinoma; PCPG, Pheochromocytoma and Paraganglioma; PRAD, Prostate adenocarcinoma; READ, Rectum adenocarcinoma; SARC, Sarcoma; SKCM, Skin Cutaneous Melanoma.

All the candidate adjuvants as well as primary analysis of genetic adjuvants can be found in AdjuvareDB (http://tmliang.cn/adjuvaredb), a user‐friendly integrated database, containing search, download and primary analysis, and etc., which may provide references for relevant studies and contribute to further clinical application. The main modules of AdjuvareDB include the home page, browser module, search module, analysis module and help module. Users can click on the module name in the navigation bar at the top of the home page to jump to the module (Figure 4), and then the database‐related introduction as well as the framework are presented. The search bar at the bottom can quickly realize the search for adjuvant‐related information. Additionally, we illustrate the clinical use of adjuvants in therapeutic cancer vaccines and summarize the application of 27 adjuvant regimens from 104 clinical trial protocols, and user can also download the adjuvants data of therapeutic cancer vaccine trials. The detailed information of adjuvants can be detected via the browse screen, mainly including protein‐based adjuvant‐related gene symbol, the detailed annotation, application in related diseases, source literatures, ingredients, and other information (Figure 4). As adjuvants are designed differently in diverse experiments, the adjuvants are often named after changes in their composition and ratios, which may confuse users with abbreviated and multi‐component combination adjuvant names. In the “More” section, the actual molecular component of an adjuvant formulation is described in detail so that users can understand the adjuvant more quickly.

**FIGURE 4:**
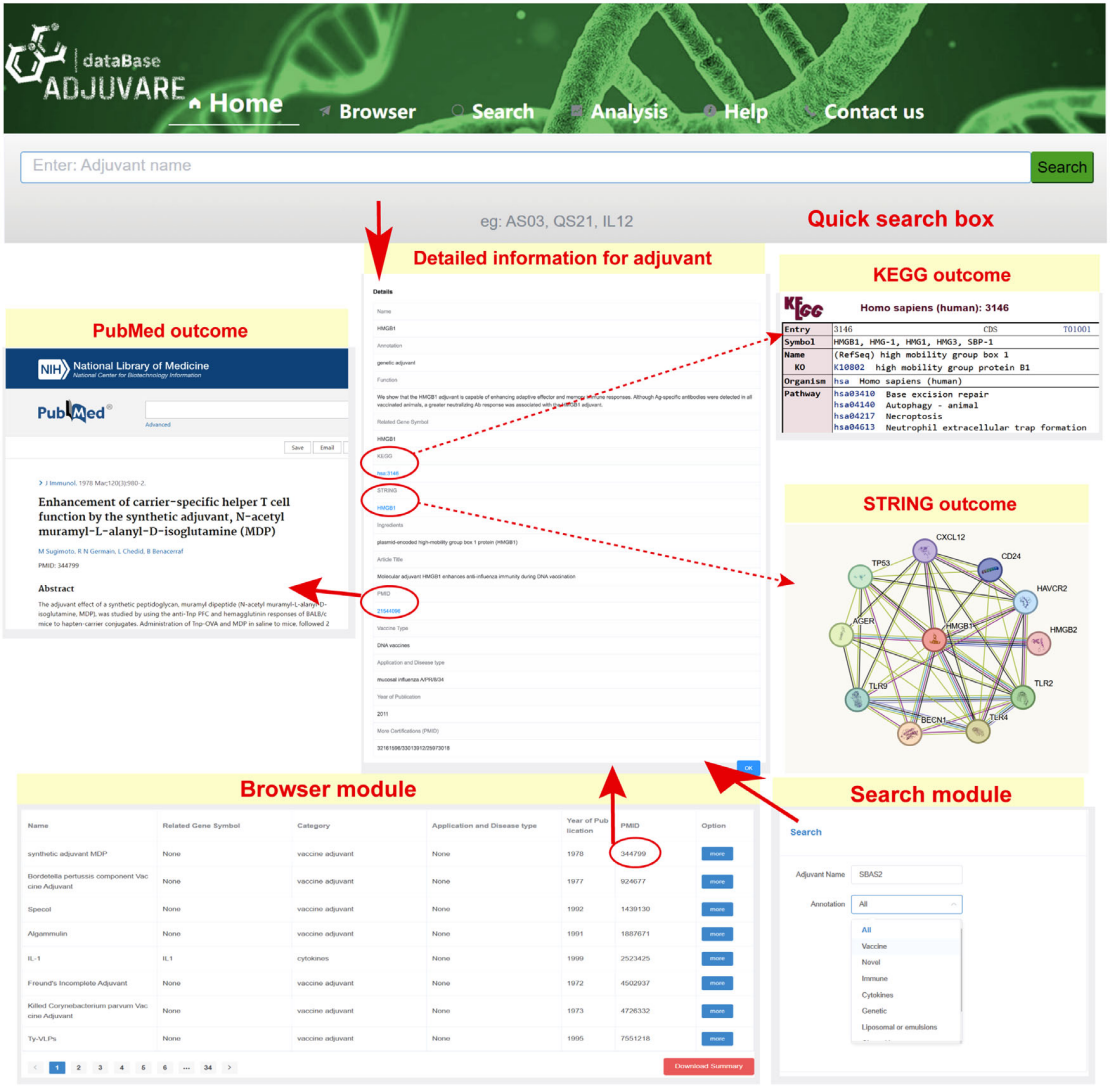
The main search module of AdjuvareDB. The main search module and the detailed information for specific adjuvant are presented here. Some candidate genetic adjuvants are also connected KEGG database and STRING database.

To provide landscapes of collected genetic adjuvants at different molecular levels that may contribute to understanding the potential molecular features in cancer, the analysis module provides the primary analysis based on integrated mRNA expression data, mutation data, DNA methylation data, copy number variation data and related clinical information (Figure S1). The Analysis interface concludes six modules, including modules of mRNA expression, mutation, methylation and copy number variation that can present alternations of genes at different molecular levels; survival module shows potential prognostic ability; and network module indicates genetic interactions based on the concept of synthetic lethality^10^ and miRNA‐mRNA regulatory network, demonstrating the potential roles of genes in tumorigenesis via a pan‐cancer analysis. Compared to similar efforts (such as the Vaxjo^8^ and Vaccine Adjuvant Compendium), we mainly extract and collate supportive literatures through text mining, but we also focus more on the potential application of adjuvants in cancer immunotherapy, providing researchers with a platform of potential adjuvant data through analysis modules and clinical trials acquisition (Figure 1).

Taken together, AdjuvareDB provides users a comprehensive compendium of existing candidate adjuvants, allowing them to compare and download information on the composition, function, and source literatures through interactive functional modules, and to further analyze the potential molecular features of candidate genetic adjuvants in pan‐cancer. Adjuvants have a wide range of applications either as vaccine components or as immune agonists in immunotherapy,^6^ and we believe that AdjuvareDB will be a valuable resource for adjuvants in vaccine and immunotherapy research and will help to further explore the most effective adjuvant composition options.

## AUTHOR CONTRIBUTIONS

Li Guo and Tingming Liang participated in the conception and design of the study. Dekang Ren, Jiaming Jin, Shizheng Xiong, Daoliang Xia, Xinmiao Zhao, Haochuan Guo, Xueni Yang, and Jiafeng Yu carried out the data collection and analyzed. Dekang Ren, Jiaming Jin, Li Guo and Tingming Liang drafted the manuscript. Dekang Ren, Li Guo and Tingming Liang revised the manuscript. All authors have approved the final version of the manuscript.

## CONFLICT OF INTEREST STATEMENT

The authors declare no conflicts of interest.

## FUNDING INFORMATION

This work was supported by National Natural Science Foundation of China (62171236), the key project of social development in Jiangsu Province (BE2022799), the key projects of Natural Science Research in Universities of Jiangsu Province (22KJA180006), the Open Research Fund of State Key Laboratory of Bioelectronics, Southeast University (SKLB2022‐K03), funding from Shandong Provincial Key Laboratory of Biophysics, Postgraduate Research & Practice Innovation Program of Jiangsu Province, and the Priority Academic Program Development of Jiangsu Higher Education Institution (PAPD).

## ETHICS APPROVAL

All authors have been personally and actively involved in substantial work leading to the paper, and will take public responsibility for its content.

## Supporting information

Supporting Information

Supporting Information

## Data Availability

AdjuvareDB (http://tmliang.cn/adjuvaredb) is freely available to the public without registration or login requirements. The data that support the findings of this study are available from the corresponding author upon reasonable request.
