# Supplementary material for: AdjuvareDB: A comprehensive database for candidate adjuvant compendium in clinic
Source: Clin Transl Med. 2024 Apr 24;14(4):e1669. [Article in Romanian] doi: 10.1002/ctm2.1669 (PMC11043087; doi:10.1002/ctm2.1669)

**Figure S1. The main analysis module of AdjuvareDB.**

For 76 candidate genetic adjuvants, they can be performed a pan-cancer analysis to understand the molecular features and association with tumorigenesis and cancer progression by a multi-omics approach.


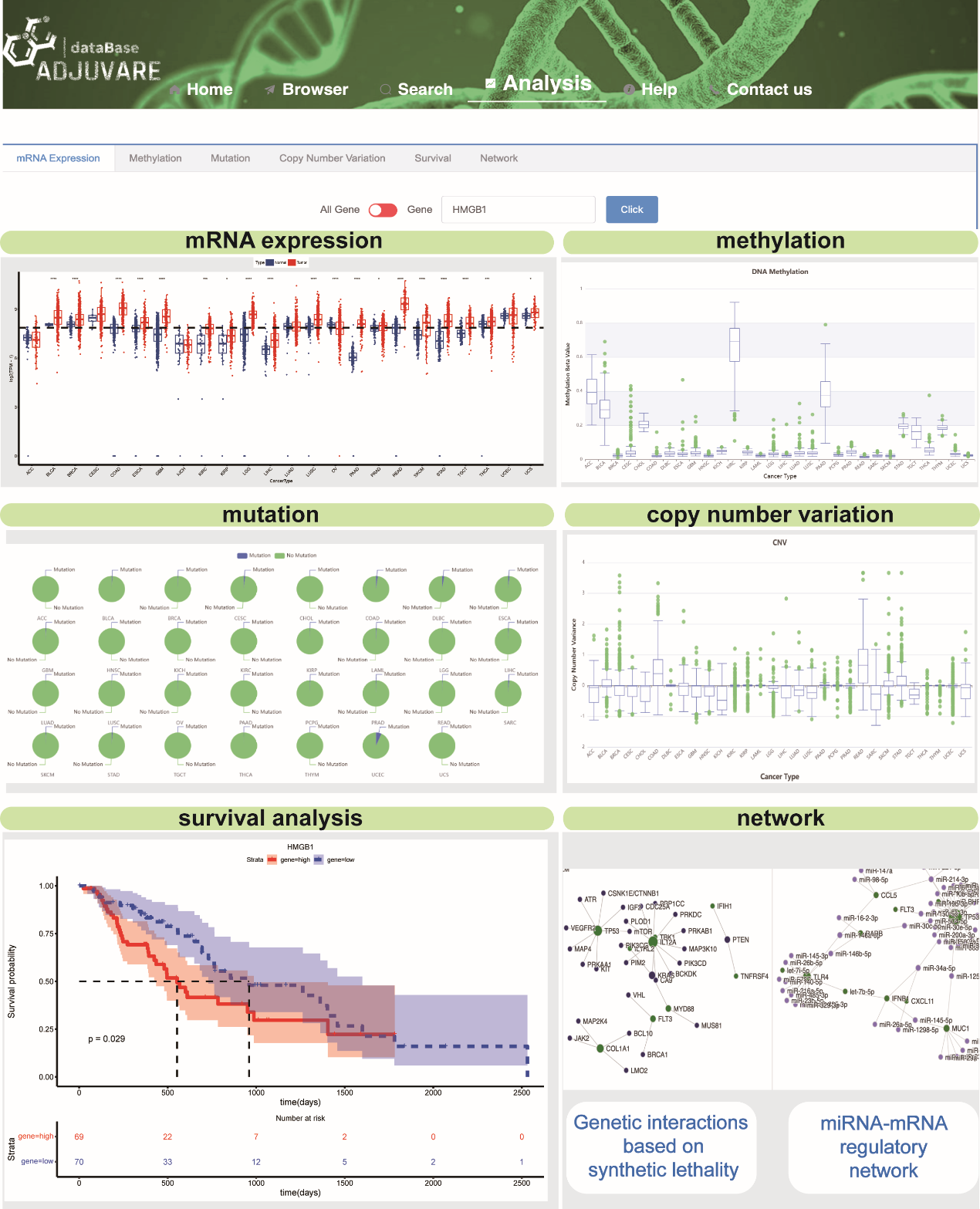

Supplement: Supplementary file 1 — Supporting Information [file CTM2-14-e1669-s002.docx]
