# Supplementary material for: AdjuvareDB: A comprehensive database for candidate adjuvant compendium in clinic
Source: Clin Transl Med. 2024 Apr 24;14(4):e1669. [Article in Romanian] doi: 10.1002/ctm2.1669 (PMC11043087; doi:10.1002/ctm2.1669)
Supplement: Supplementary file 2 — Supporting Information [file CTM2-14-e1669-s001.docx]

**Materials and methods**

**Data collection**

To provide the most comprehensive information on adjuvants, reported adjuvants were collected based on published papers, existing databases, and several websites for relevant adjuvants in vaccines and novel materials as adjuvants in delivery systems (Figure 1A). We preliminarily identified several groups of keywords related to adjuvants, mainly including immune adjuvants, genetic adjuvants, vaccine adjuvants and immunoadjuvants, and these keywords were used to conduct a literature search on PubMed (http://www.ncbi.nlm.nih.gov/pubmed), eliminating the literature types of reviews and case studies, and then proceeding to the remaining article types were searched, and finally about 10,400 relevant literature that met the search criteria were obtained.

According to collected relevant articles and adjuvants, the relevant contents were summarized, including source literature, year, type of vaccine to which they belonged, the direction of application, composition, function and effect. Based on the integration of information from existing adjuvant-related research, we have compiled a variety of vaccine design options for cancer vaccines, such as mRNA vaccines, peptide vaccines, and dendritic cell-based vaccines. Simultaneously, compared with adjuvant database Vaxjo (http://www.violinet.org/vaxjo/)[1], another 14 adjuvants in Vaxjo were also obtained. Among these collected adjuvants, we noticed potential genetic adjuvants such as pattern recognition receptors, playing a key role in immune activation and enhancement. By querying these genetic adjuvants through GeneCards (GeneCards®: The Human Gene Database, https://www.genecards.org/)[2], we obtained 76 potential protein-related adjuvant target genes.

Finally, 331 adjuvants were screened and obtained, which were presented in AdjuvareDB as well as the primary analysis via separate modules.

**Primary analysis of molecular alternations in pan-cancer**

Among collected 331 adjuvants, some were identified as related genes in adjuvant components, and these genes may be potential adjuvants. For example, the genetic adjuvant IL-12 can enhance the protective efficacy of a DNA vaccine for Venezuelan equine encephalitis virus delivered by intramuscular injection in mice [3], pyroptosis-inducing active caspase-1 may act as genetic adjuvant in anti-cancer DNA vaccination [4], and genetic adjuvant therapy for pancreatic cancer and other solid tumors may be a promising approach [5]. Abnormal and uncontrolled cell growth caused by genetic mutations drives cancer progression, such as tumor mutation burden (TMB) as an immunotherapy biomarker [6]. Since tumors with high TMB also have a greater number of neoantigens, suggesting that tumors with high TMB have a stronger immunogenicity heel suitable for cancer vaccines as an immunotherapeutic strategy [7, 8]. Therefore, based on the potential clinical application in anti-tumor treatment as well as the critical roles in pathological and physiological processes, these genes were also performed pan-cancer analysis to understand the potential molecular features in tumorigenesis and cancer progression, mainly including alternation at multiple levels, mRNA, mutation, DNA methylation and copy number variation, based on retrieved data from The Cancer Genome Atlas (TCGA) and Genotype-Tissue Expression (GTEx). Specifically, expression analysis was performed using the limma package [9], and expression distributions for specific genes can be obtained from the AdjuvareDB. Mutation profiles and CNV were also presented. Further, based on expression data, survival analysis was performed to understand the potential prognostic values in pan-cancer using survminer and survival packages. Generalized subsystem vibrational analysis (GSVA) was also performed to estimate the potential functional implication using the R package GSVA [10] and GSCA platform [11].

Moreover, in order to understand the potential roles of genes as genetic adjuvants in cancer treatment, these genes were also queried for the potential genetic interactions with other genes based on the concept of synthetic lethality [12] according to experimentally validated interactions [13-20]. Then, according to the important regulatory roles of small ncRNAs (non-coding RNAs), microRNAs (miRNAs), miRNA-mRNA regulatory network was also constructed and presented according to experimentally validated regulatory relationships [21], which would contribute to understanding the potential regulatory roles of flexible miRNAs.

**Web interface implementation**

AdjuvareDB is a MySQL database developed utilizing the Spring architecture (Figure 1B). The database was designed with a front-end and back-end separation approach, where the back-end contains the data access layer consisting of Springboot, and the front-end implements the Vue framework for its presentation layer. The front-end and back-end communicate used HTTP (Hypertext Transfer Protocol) and exchange data in JSON (JavaScript Object Notation) format. Finally, the presentation layer utilized the R language to provide analysis services, and the "Rserve" package constructed the server enabling Java to execute R scripts for processing analysis requests.

**References**

1. Sayers S, Ulysse G, Xiang Z, He Y: **Vaxjo: a web-based vaccine adjuvant database and its application for analysis of vaccine adjuvants and their uses in vaccine development**. *J Biomed Biotechnol* 2012, **2012**:831486.

2. Safran M, Solomon I, Shmueli O, Lapidot M, Shen-Orr S, Adato A, Ben-Dor U, Esterman N, Rosen N, Peter I *et al*: **GeneCards 2002: towards a complete, object-oriented, human gene compendium**. *Bioinformatics* 2002, **18**(11):1542-1543.

3. Suschak JJ, Bagley K, Six C, Shoemaker CJ, Kwilas S, Spik KW, Dupuy LC, Schmaljohn CS: **The genetic adjuvant IL-12 enhances the protective efficacy of a DNA vaccine for Venezuelan equine encephalitis virus delivered by intramuscular injection in mice**. *Antiviral research* 2018, **159**:113-121.

4. Arakelian T, Oosterhuis K, Tondini E, Los M, Vree J, van Geldorp M, Camps M, Teunisse B, Zoutendijk I, Arens R *et al*: **Pyroptosis-inducing active caspase-1 as a genetic adjuvant in anti-cancer DNA vaccination**. *Vaccine* 2022, **40**(13):2087-2098.

5. Bagley KC: **Genetic adjuvant therapy for pancreatic cancer and other solid tumours**. *Gut* 2008, **57**(3):289-291.

6. Morad G, Helmink BA, Sharma P, Wargo JA: **Hallmarks of response, resistance, and toxicity to immune checkpoint blockade**. *Cell* 2021, **184**(21):5309-5337.

7. Cristescu R, Mogg R, Ayers M, Albright A, Murphy E, Yearley J, Sher X, Liu XQ, Lu H, Nebozhyn M *et al*: **Pan-tumor genomic biomarkers for PD-1 checkpoint blockade-based immunotherapy**. *Science* 2018, **362**(6411):114-138.

8. Chan TA, Yarchoan M, Jaffee E, Swanton C, Quezada SA, Stenzinger A, Peters S: **Development of tumor mutation burden as an immunotherapy biomarker: utility for the oncology clinic**. *Ann Oncol* 2019, **30**(1):44-56.

9. Ritchie ME, Phipson B, Wu D, Hu Y, Law CW, Shi W, Smyth GK: **limma powers differential expression analyses for RNA-sequencing and microarray studies**. *Nucleic Acids Res* 2015, **43**(7):e47.

10. Hanzelmann S, Castelo R, Guinney J: **GSVA: gene set variation analysis for microarray and RNA-seq data**. *BMC Bioinformatics* 2013, **14**:7.

11. Liu CJ, Hu FF, Xia MX, Han L, Zhang Q, Guo AY: **GSCALite: a web server for gene set cancer analysis**. *Bioinformatics* 2018, **34**(21):3771-3772.

12. Huang A, Garraway LA, Ashworth A, Weber B: **Synthetic lethality as an engine for cancer drug target discovery**. *Nat Rev Drug Discov* 2020, **19**(1):23-38.

13. Lee JS, Das A, Jerby-Arnon L, Arafeh R, Auslander N, Davidson M, McGarry L, James D, Amzallag A, Park SG *et al*: **Harnessing synthetic lethality to predict the response to cancer treatment**. *Nature communications* 2018, **9**(1):2546.

14. Neggers JE, Paolella BR, Asfaw A, Rothberg MV, Skipper TA, Yang A, Kalekar RL, Krill-Burger JM, Dharia NV, Kugener G *et al*: **Synthetic Lethal Interaction between the ESCRT Paralog Enzymes VPS4A and VPS4B in Cancers Harboring Loss of Chromosome 18q or 16q**. *Cell reports* 2020, **33**(11):108493.

15. Srivas R, Shen JP, Yang CC, Sun SM, Li J, Gross AM, Jensen J, Licon K, Bojorquez-Gomez A, Klepper K *et al*: **A Network of Conserved Synthetic Lethal Interactions for Exploration of Precision Cancer Therapy**. *Molecular cell* 2016, **63**(3):514-525.

16. Parrish PCR, Thomas JD, Gabel AM, Kamlapurkar S, Bradley RK, Berger AH: **Discovery of synthetic lethal and tumor suppressor paralog pairs in the human genome**. *Cell reports* 2021, **36**(9):109597.

17. Najm FJ, Strand C, Donovan KF, Hegde M, Sanson KR, Vaimberg EW, Sullender ME, Hartenian E, Kalani Z, Fusi N *et al*: **Orthologous CRISPR-Cas9 enzymes for combinatorial genetic screens**. *Nature biotechnology* 2018, **36**(2):179-189.

18. Shen JP, Zhao D, Sasik R, Luebeck J, Birmingham A, Bojorquez-Gomez A, Licon K, Klepper K, Pekin D, Beckett AN *et al*: **Combinatorial CRISPR-Cas9 screens for de novo mapping of genetic interactions**. *Nature methods* 2017, **14**(6):573-576.

19. Guo J, Liu H, Zheng J: **SynLethDB: synthetic lethality database toward discovery of selective and sensitive anticancer drug targets**. *Nucleic acids research* 2016, **44**(D1):D1011-1017.

20. Li XJ, Mishra SK, Wu M, Zhang F, Zheng J: **Syn-lethality: an integrative knowledge base of synthetic lethality towards discovery of selective anticancer therapies**. *BioMed research international* 2014, **2014**:196034.

21. Huang HY, Lin YC, Cui S, Huang Y, Tang Y, Xu J, Bao J, Li Y, Wen J, Zuo H *et al*: **miRTarBase update 2022: an informative resource for experimentally validated miRNA-target interactions**. *Nucleic Acids Res* 2022, **50**(D1):D222-D230.
